# Gunshot Wound of the Thoracic Aorta with Right Popliteal Artery Embolization: A Case Report of Bullet Embolism with Review of Relevant Literature

**DOI:** 10.1155/2013/198617

**Published:** 2013-04-11

**Authors:** Saptarshi Biswas, Hadley Cadot, Sunil Abrol

**Affiliations:** Department of General Surgery, Brookdale University Hospital Medical Center, Brooklyn, NY 11212, USA

## Abstract

Bullet embolism is a well-known but relatively uncommon complication of gunshot injuries. Their rarity and the potential lack of early symptoms lead to delays in diagnosis and often in inadequate early management that can potentially result in the loss of a limb or life. We present an interesting case in which a small caliber bullet to the upper anterior abdomen penetrated the thoracic aorta and traveled to the right popliteal artery embolizing the vessel. The exploratory laparotomy failed to locate neither the bullet nor the trajectory resulting in sudden deterioration and eventual death 5 hours into the postoperative period.

## 1. Introduction

Bullet emboli after vascular trauma remain an unusually rare yet fascinating entity. The increased incidence of gun violence in a civilian urban setting has increased the possibility of encountering bullet embolism. This is particularly applicable with relatively low velocity missiles because of the fact that most of the kinetic energy has been dissipated by entering the blood vessels, and the flow of the bloodstream sweeps the bullet away often to a distant location resulting in embolisation.

Many such emboli are notoriously asymptomatic and thus can be missed on initial evaluation. Whenever initial workup fails to visualize the bullet or the entry and exit wounds do not match up, a detailed and often remote imaging may need to be obtained provided the patient remains hemodynamically stable. Unexplained trajectories of bullets should trigger the suspicion of bullet embolism.

## 2. Case Report

A 20-year-old African-American male was brought in as an activated trauma by an emergency medical service. He was status post gunshot wound to the left upper abdomen. The gunshot wound occurred while the victim was facing an unidentified attacker. The weapon was unidentified. Although the precise time of injury was unknown, according to the emergency medical service they were immediately notified, and the patient arrived to the hospital within minutes of the incident. 

The patient was alert and oriented to person, place, and time. He appeared distinctly uncomfortable but in no obvious distress and answering questions appropriately. Vitals recorded in the trauma bay read as T 37.2 C, HR118, BP110/70, and RR 28 saturating 99% on face mask.

Primary survey revealed cleared airway; lungs were clear with bilateral air entry. His vitals on admission were within stable limits. All peripheral pulses were palpable and symmetrical. Brief neurological examination showed GCS 15 moving all his extremities with gross intact sensory and motor functions. Two large bore intravenous access lines were established on Ringer's lactate. A thorough examination of the torso revealed a solitary bullet wound in the left upper quadrant of the anterior abdominal wall. There was no obvious active bleeding noted from his wound. There was no other “exit” wound noticed on secondary survey. A chest and pelvis X-rays were ordered as adjunct of the primary workup.

His past medical history was significant for lymphoma, and he was in remission after chemotherapy. His past surgical history includes two exploratory laparotomies. One was for a prior gunshot wound and the other for a knife injury. He denied any drug or other allergies. He did not provide a social or personal history at the time of the admission. 

**Figure 1:**
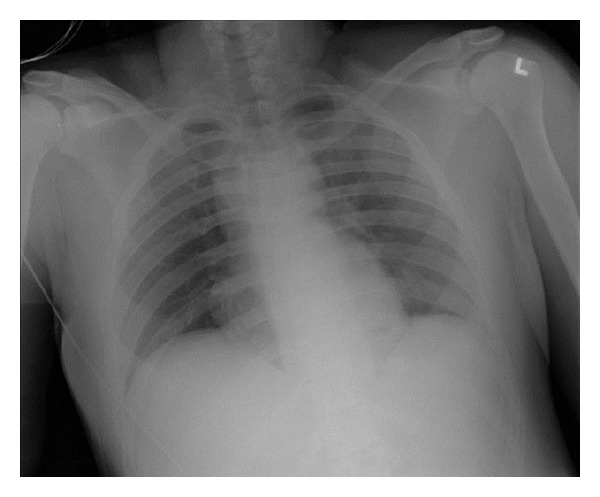
An AP chest X-ray in the trauma bay showed no obvious pneumothorax, hemothorax, or bullet fragment.

**Figure 2:**
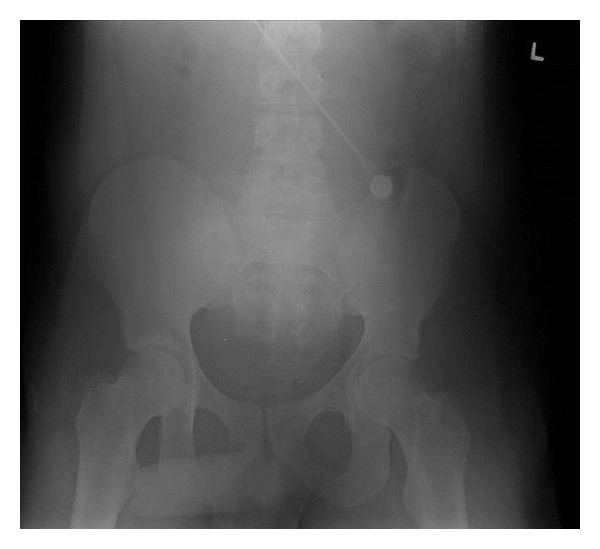
Pelvic X-ray did not reveal any fracture or radiopaque foreign body.

Upon admission, his labs were as follows: WBC, hemoglobin 12.2, hematocrit 36.4, platelets 202, K 3.5, Na^+^ 164, CO_2_ 19, Cl 106, BUN 24, creatinine 2.0, protein 6.1, albumin 3.7, Ca^++^ 8.3, ALT 17, and AST 64 (Figures 1 and 2).

Because of the high velocity penetrating nature of the injury, a decision was made to take the patient for an exploratory laparotomy. Upon insertion of a Foley catheter, there was no gross hematuria. Extensive abdominal adhesions made exploration of the abdomen time consuming. Care was taken not to cause any inadvertent injury to underlying bowel or other viscera. Upon entering the abdomen, there was presence of some blood in the peritoneum. However, no gross evidence of zone I or II active bleeding or hematoma was noted. After removal of 4 quadrant packing, there was some diffuse oozing in the left upper quadrant. There was no obvious splenic injury, with the splenic capsule intact. Some bleeding appeared from the region of short gastric vessels into the splenic bed. A through-and-through injury to the cardia was identified. The anterior stomach wound was sutured in layers, and the posterior wound was closed with gastrointestinal anastomotic stapler device. A 5 cm liver laceration on the anterosuperior aspect was sutured with cat gut blunt liver sutures. One isolated small bowel enterotomy in the left upper quadrant was repaired with interrupted sutures. Topical coagulants Fibrillar and Aventine were applied for local hemostatic control of diffuse oozing in the left upper quadrant. Intraoperatively, patient received four units of packed red blood cells and two units of fresh frozen plasma. Once we were convinced that hemostasis has been secured, a decision was made to close the abdomen and resuscitate.

No bullet/fragment was found during the process of the surgery nor any retroperitoneal second wound. The patient was transferred to the postanesthesia care unit in critical, but stable condition. Throughout the next 5 hours, he continued to be relatively stable with PH improving from 7.20 to 7.24 to 7.29. His HCO3 was getting better from 13.2 to 14.5 to 16.7. So although he was still in metabolic acidosis still undergoing active resuscitation, his base deficit was progressively getting better from an immediate postoperative value of 13.7 down to 9.0 within the next 5 hours.

**Figure 3:**
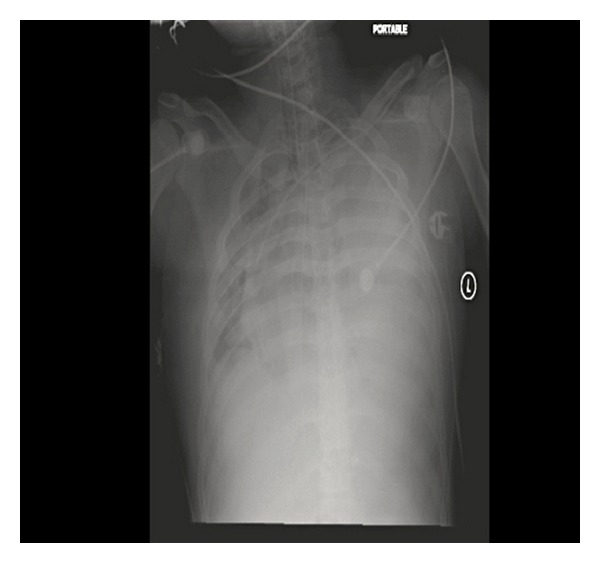
Chest X-ray 5 h after surgery demonstrates complete opacification of the left hemothorax with shift of the mediastinum to the right.

**Figure 4:**
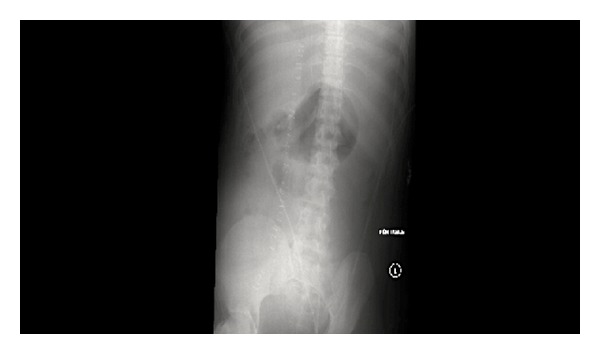
Single supine film of the abdomen at 5 h after surgery demonstrates a left upper quadrant opacity. no bullet is seen.

Approximately 5 hours after surgery, the patient suddenly became hypotensive with a systolic blood pressure plunging to as low as the 60 s. The patient was transfused with PRBC and FFPs. Norepinephrine drip was started followed by neosynephrine. They were quickly titrated up to a maximum level. In spite of the administration of multiple pressors and blood products, the decompensation was swift and the patient went into asystole arrest. Advanced cardiovascular life support proved futile, and the patient was pronounced. After the futile attempt to revive the patient, X-rays of the chest and abdomen were performed to throw some light into the cause of death of the patient who till then had shown progressive signs of resuscitation with a declining base deficit improving PH and bicarbonate level (Figures 3 and 4).

The medical examiner's report documented significant bleeding into the chest with the accumulation of 2 L of blood in the left chest and 1 L in the right chest. There was one bullet injury at the level of T10. It went from the left superior-anterior abdomen through and through the body of the stomach. It then penetrated the thoracic aorta and embolized to subsequently lodge in the right popliteal artery. The caliber of the bullet as per the autopsy report was 0.22.

## 3. Discussion

Bullet embolism within the vascular stream is a rare consequence of penetrating gunshot injuries [1, 2]. Historically the first bullet embolism was reported by Thomas Davis in 1834 and later cited by Sir Bland-Sutton in 1919 [3]. A study by Rich et al. [4] of 7500 GSW wounds during Vietnam War revealed only 22 cases complicated by foreign body emboli (0.3%). A 10-year retrospective review by Sandler et al. [5] revealed only 46 cases of GSW wounds resulting in missile embolism. Earlier Adegboyega et al. in Journal of Trauma [6] in 1996 reported that since 1834 only 160 cases of bullet embolism were reported.

Slobodan et al. [1] mention that more than 70% of cases of missiles penetrate into the arterial circulation through either the thoracic or abdominal aorta or even through the heart [1, 7–11]. However, there have been isolated reports of distal arterial embolization from peripheral arteries [12, 13]. The embolism is antegrade in majority of cases. Rare cases of retrograde (venovenous) [14–16] and paradoxical [17] embolism are also reported. Schmelzer et al. [16] and Rich et al. [4] suggest that arterial missile emboli outnumber venous ones 4 : 1. In our case, the autopsy study revealed that the bullet entered through the thoracic aorta, entering the systemic circulation and eventually lodging into the right popliteal artery. The embolism in our case might have occurred shortly after the initial injury or in the perioperative resuscitative phase. However, there have been a number of case reports [18–21] when the embolisation was delayed for days, weeks, or longer. Wales et al. [22] have reported a unique case of RV bullet embolism that manifested itself clinically 4 years after initial insult.

Patel et al. [18] have explained the low incidence of bullet embolism. For a projectile to become an embolus, 2 major prerequisites needed to be satisfied. First, the bullet should have little kinetic energy remaining at the precise instant it enters the blood vessel. Second, the diameter of the bullet must be less than the diameter of the blood vessel it penetrates. This explains why the incidence of bullet embolism is so low in war scenarios. They reported an incidence of 55% with 0.22 caliber handgun and 27% with shotgun.

Small caliber bullets are more prone to tumbling, may not pierce blood vessels, have a slower velocity, and are able to fit through peripheral blood vessels which are too narrow for large caliber bullets. The gun and ammunition used in the trauma are also important to identify, because bullet emboli are more common with smaller, blunt nose, short length, or low velocity bullets. Bullet fragments or pellets are also more prone to embolization.

This explains the scenario in our case where a 0.22 caliber was fired probably from a distance of 15–20 feet, so that the kinetic energy was sufficient to penetrate the thoracic aorta but not enough to exit it. The bullet was also small enough to migrate to the right lower extremity following the blood stream and eventually embolizing the right popliteal artery.

In cases in which a bullet is not immediately located, it is important to determine the initial bullet trajectory before its path was possibly changed in the body. To determine this, first the relation of the location of the victim to the shooter must be determined. Then, the axis of the gun barrel must be determined.

Factors postulated as responsible for determining distant lodgment sites are as follows: (1) power of the missile, (2) caliber and shape, (3) site of penetration into the vascular bed, (4) effect of gravity especially in the low pressure venous system, (5) respiratory activity, (6) force of blood flow, (7) position of the victim at the moment of penetration of the embolus into the circulation, and (8) relative size and angle of the arterial branches [5, 18, 23, 24].

Shannon et al. [13] reported a 76% rate to lower extremities with the left side affected twice as often as the right. The authors postulated that the left common iliac artery branches from the aorta at a less acute angle than the right common iliac artery and accounts for the difference in incidence [23]. The findings by Sandler et al. were almost evenly distributed in either lower extremities (5 on the left and 4 on the right). Shen et al. [11] reviewed projectile embolus to the lower extremity after penetrating thoracic aortic injury. Of the 14 cases reviewed, the incidence of left and right sided embolus was equal.

Although the final lodgement site remains mysteriously unpredictable, some common patterns are noticed. A peripheral venous embolism usually terminates in the right heart or pulmonary artery. A right heart bullet goes to the pulmonary artery. Shotgun pellets or bullet fragments originating from the left heart usually embolize to the middle cerebral arteries. The reports show a 70% predilection for the right brain compared to the left (30%) [18, 25]. The explanation can be attributed to the flow patterns into the first and largest branch of the aortic arch, the innominate artery.

Penetrating injury to the thoracic aorta usually carries a dismal prognosis with only 20% of patients making it to the hospital [26]. Demetriades et al. have quoted a 92% mortality rate for injuries to the thoracic aorta [27]. Shen et al. [11] have done a comprehensive review of gunshot wound to the thoracic aorta with peripheral arterial bullet embolism. He reported 18 documented cases between 1960–1980 found in an English language Medline search. Shannon et al. [13] reviewed 30 cases of peripheral arterial missile embolisation in a 22-year study period. He found that approximately 33% of those patients had a thoracic aortic site of entrance.

Provided that the initial insult does not prove fatal, arterial projectile embolus has a considerably significant consequences compared to a venous embolism. In the series by Sandler et al. [5], there was a reported incidence of 65% with signs or symptoms of ischemia. The same article mentions the proportion of complicated arterial to venous emboli as 3 : 1. The failure to diagnose and remove a missile embolus in the arterial system may result in significant irreversible damage.

Missile embolism not only causes acute ischemia or infarction but also may result in displacement, erosion, embolization further into the vascular tree, delayed vascular compromise, proximal clot formation, or septicemia, or even lead to intoxication and even death [6, 16]. Schwoerer et al. [28] describe an incidence of 66.7% of peripheral ischemia in patients who presented with arterial peripheral embolism. The same report mentions that 13.3% present with neurological deficits and 20% remain asymptomatic in the same patient group. All projectiles can potentially carry bacteria. Besides, they can also carry into the wound skin flora, clothing, and foreign particles any of which can act as a nidus of infection independently [29]. Bilsker et al. [30] have reported a unique case clostridium difficile subacute bacterial endocarditis from a bullet penetrating the left ventricle and subsequently to the right subclavian artery. Cardiac bullet embolus can cause cardiac irritability, delayed embolism [16, 31, 32], and recurrent pericardial effusions and may even interfere with valvular functioning [33].

A very high index of suspicion is of utmost importance to identify a missile embolus. If the number of entry wounds does not equal the number of exit wounds or the clinical signs or symptoms and radiologic imaging do not correlate with the injury, the possibility of bullet embolism should be thought of. Unexplained peripheral ischemia, sudden loss of peripheral pulse, or abrupt onset of new unexpected neurologic findings should trigger the suspicion of missile embolism. X-rays, CT scans, USG, echocardiography, and angiography are used as diagnostic tools. MRI should be avoided in case the projectile has a ferrous component [5].

Symptomatic peripheral and visceral emboli should be removed by intervention radiology or open surgical method. In selected situations where it has already caused visceral or neurological infarction and unlikely to cause any further harm, the embolus can be left in place. If the risk of the intervention outweighs the potential benefits of removing the embolus, it is advisable to play safe rather than cause any additional iatrogenic damage.

## 4. Conclusion

This case demonstrates how a bullet initially unaccounted for influences the course and outcome of a trauma patient. Given the grave outcome of penetrating thoracic aorta injury, an additional confounding variable such as a migratory arterial bullet presents a truly formidable challenge for even the most experienced trauma surgeon. This case emphasizes that the true path of a bullet or fragment cannot be always ascertained based on the site of the entrance wound. However, when the suspicion of a bullet embolism has been raised, the missing projectile should be thoroughly accounted for by a thorough and meticulous search and the resultant damage addressed even if the patient is asymptomatic on initial presentation. The consequence of a missed bullet embolism may prove fatal as our case taught us.
